# Diagnosis and Management of a Unique Iatrogenic Biatrial Gerbode Defect

**DOI:** 10.1155/2022/6998632

**Published:** 2022-07-18

**Authors:** Karim Shuaib, Giacomo Scorsese, Christopher Seiter, Eric Zabirowicz, Jeremy Poppers

**Affiliations:** Department of Anesthesiology, Stony Brook University Health Science Center, Stony Brook, NY 11794-8480, USA

## Abstract

The Gerbode defect was first described in the late 1950s as a congenital peri-membranous ventricular septal defect (VSD), resulting in a left to right ventriculoatrial shunt. We present a case of a patient with restenosis of a prior bioprosthetic aortic valve (AV) who underwent reoperative AV replacement (AVR), which was complicated by a unique iatrogenic Gerbode defect with concurrent LV-LA communication. Our case highlights the unique complications resulting from ventriculoatrial shunts, with consideration paid to the management of ventriculoatrial defects described.

## 1. Introduction

The Gerbode defect was first described in 1958 as a congenital peri-membranous ventricular septal defect (VSD), resulting in a left to right ventriculoatrial shunt [1]. Although still rare, reports of an acquired Gerbode left ventricular (LV) to right atrial (RA) shunt have become increasingly frequent with the exponential rise in complex invasive cardiac procedures [2].

We present a case of a patient with restenosis of a prior bioprosthetic aortic valve (AV) who underwent reoperative AV replacement (AVR), which was complicated by a unique iatrogenic Gerbode defect with concurrent LV-LA communication. Our case will highlight the pathophysiology, as well as the diagnostic, hemodynamic, and therapeutic options for this Gerbode variation.

## 2. Case Presentation

A 77-year-old woman with a past medical history significant for aortic stenosis who had undergone surgical AVR (21 mm trifecta, St. Jude Medical, Saint Paul, MN) seven years prior was admitted to our hospital with worsening dyspnea on exertion. A transthoracic echocardiogram (TTE) demonstrated significant stenosis of the bioprosthetic AV with severe restriction of the anatomic non- and left-coronary cusps with peak and mean gradients of 88 and 55 mmHg, respectively, and a dimensionless index of 0.21. Other significant findings on the TTE included normal left ventricular (LV) function, moderately reduced right ventricular function (tricuspid annular plane systolic excursion of 1.43 cm), moderate mitral valve (MV) regurgitation, trace tricuspid regurgitation, and an estimated pulmonary artery systolic pressure of 51 mmHg. The interatrial septum was also noted to be intact. The patient was deemed anatomically unsuitable for a transcatheter AVR approach and was therefore scheduled to undergo a preoperative surgical AVR. Given the patient's advanced age and poor functional status, as well as to minimize what was expected to be a prolonged cardiopulmonary bypass (CPB) and cross-clamp time, the operative plan was to address the mitral regurgitation in the future by means of a percutaneous minimally invasive procedure.

The patient's initial transesophageal echocardiogram (TEE) confirmed the preoperative TTE findings related to the AV pathology in the operating room. The TEE also demonstrated normal biventricular function, mild-moderate central MR, moderate-severe TR with an estimated annular measurement of 4.2 cm, and a dilated right atrium of 4.7 cm. The patient was placed on CPB via right axillary artery and right femoral vein access, the aorta was cross-clamped, and the heart was arrested in standard fashion. During excision of the diseased bioprosthetic valve, extensive calcifications were also noted to involve the base of the anterior MV leaflet. Thus, in addition to the planned insertion of a 19 mm Inspiris bioprosthetic AV (Edwards Lifesciences, Irvine, CA), multiple sutures were placed to reinforce the aorto-mitral curtain, and a 28 mm physio ring (Edwards Lifesciences, Irvine, CA) was inserted in the tricuspid position. The entire process of dissection, debridement, and reconstruction was completed during a 2-hour and 24-minute aortic cross-clamp time. Upon weaning from CPB, the patient was hemodynamically unstable despite using multiple inotropic and vasopressor agents. The post-bypass TEE was notable for normal LV function, mild-moderate RV dysfunction with significant central MR, and an additional regurgitant jet at the base of the anterior MV leaflet. It was then decided to reinstitute CPB and address the mitral valve by placing additional sutures at the base of the anterior leaflet, an Alfieri stitch between the A2-P2 segments, and stabilize the annulus with a 28 mm Cosgrove annuloplasty band (Edwards Lifesciences, Irvine, CA). Inhaled nitric oxide and milrinone were initiated, and a 7fr intra-aortic balloon pump (IABP) was inserted to facilitate separation from CPB. The patient's chest was left open due to concerns over surgical bleeding, elevated pulmonary artery pressures, and hemodynamic instability.

**Figure 1:**
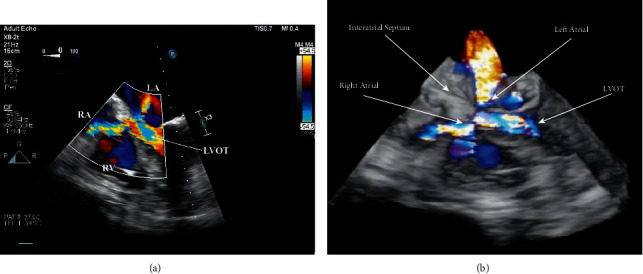
Postoperative day 2 TEE demonstrating two distinct regurgitant jets by color flow Doppler (CFD) with a common origin in the LVOT communicating with both the RA and LA. (a) 2D CFD of the mid-esophageal four-chamber view that has been cropped and (b) 3D CFD full-volume reconstruction (Philips X8-2t probe, Cambridge, MA).

**Figure 2:**
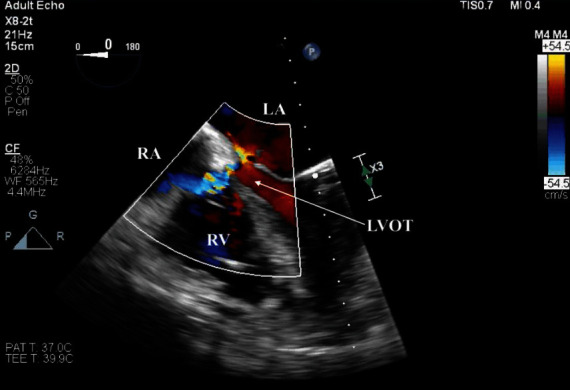
2D mid-esophageal four-chamber view with CFD demonstrating biatrial regurgitant jets originating in the LVOT and occurring into diastole (Philips X8-2t probe, Cambridge, MA).

**Figure 3:**
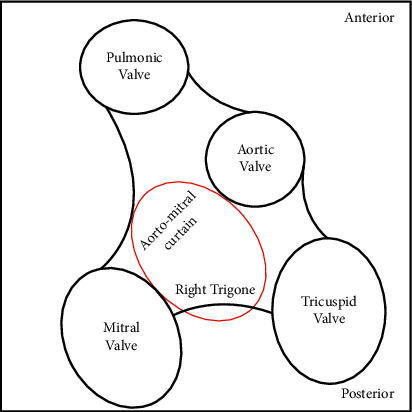
En face representation of the intervalvular fibrous trigone and the likely location of the injury to the area of the right fibrous trigone (red circle) that is in continuity with the LVOT and the inferior portion of the interatrial septum (Figure 1).

Due to continued hemodynamic deterioration secondary to progressive cardiogenic shock, the patient was again taken back to the operating room on postoperative day (POD) 2 for a washout and insertion of a right axillary Impella 5.0 device (AbioMed, Danvers, MA). TEE performed during this time, utilizing 2D and 3D full-volume reconstruction with color flow Doppler overlay (Philips X8-2t probe, Cambridge, MA), demonstrated two distinct color flow jets occurring during systole (Figure 1) and, to a lesser extent, diastole (Figure 2) with a common origin in the LV outflow tract (LVOT) that communicated with both the RA and LA. These diverging jets likely occurred iatrogenically during the excision of the prior bioprosthetic AV and debridement of the extensive calcifications that extended from the valve to the surrounding anatomy. The perforation originated in the LVOT and violated the right fibrous trigone and the inferior interatrial septum (Figure 3). After successfully inserting a right axillary Impella 5.0 device, the patient's chest could be closed without further hemodynamic compromise. However, she was transported up to CTICU in critical condition on high-dose vasopressor and inotropic support (i.e., norepinephrine, vasopressin, phenylephrine, milrinone, and epinephrine).

Over the following day, her clinical course continued to decline despite maximal medical therapy and mechanical support from both her Impella 5.0 and IABP. By POD 3 the patient had deteriorated into a state of severe shock, renal failure, liver failure resulting in coagulopathy, and suprasystemic PA pressures despite inhaled nitric oxide at 20 ppm. With no apparent viable solution to her structural heart defect, the family ultimately decided to withdraw support.

## 3. Discussion

The Gerbode defect is an extremely rare form of congenital peri-membranous VSD, resulting in a left to right ventriculoatrial shunt [2]. Acquired ventriculoatrial shunts have been reported to occur as a complication of myocardial infarction/necrosis, endocarditis, and cardiac surgery, particularly aortic and mitral valve interventions [2, 3].

With Gerbode defects, shunting occurs from the LV to RA, resulting in RA volume and pressure overload and concomitant symptoms of dyspnea and heart failure. A prominent pansystolic murmur can be appreciated along the left sternal border on physical exam [2]. TEE is the diagnostic procedure of choice [4]. Color flow Doppler will identify a high-velocity systolic jet with flow directed toward the RA which may be present, albeit diminished throughout diastole. Silbiger [5] specified several key echocardiographic clues suggestive of the Gerbode defect, including (a) atypical jet direction, (b) persistent shunt flow into diastole, (c) lack of ventricular septal flattening, (d) absence of right ventricular hypertrophy, and (e) normal diastolic pulmonary artery pressure as estimated from the pulmonic regurgitant velocity.

Our case offers additional complexity with the concomitant LV-LA communication through an injury from the LVOT through the right fibrous trigone and the inferior interatrial septum. This rare defect has previously been described in isolation secondary to inflammatory degradation or iatrogenic causes, resulting in LA volume and pressure overload, dyspnea, and heart failure [6, 7].

The hemodynamic goals for the Gerbode defect would be to reduce systemic vascular resistance, increasing cardiac output and diminishing pulmonary congestion [8]. A modest increase in HR may aide in handling the extra chamber volume. An intra-aortic balloon counter pulsation device can be used as a temporizing measure [9]. In addition, use of a percutaneous transaortic left ventricular assist device may improve forward cardiac output.

Dysrhythmias, especially atrial fibrillation, can arise from structural changes that develop with increased atrial pressures [10]. Preserving native or supporting with temporary atrioventricular pacing is critically important in patients with these defects to promote adequate LV filling, stroke volume, and forward flow in the setting of severe regurgitant blood flow and heart failure symptoms.

Definitive therapy may be achieved in the interventional cardiology suite with use of an Amplatzer occluder device [1, 11]. Alternatively, a high-risk open surgical option known as the commando procedure, which consists of intervalvular fibrous body repair with mitral and aortic valve replacement, may also be attempted [12]. If the shunt fraction does not contribute to any hemodynamic instability, it may be prudent to avoid intervention of any kind.

## 4. Conclusion

Our case introduces a unique iatrogenic injury resulting in a combination of a Gerbode defect and an LVOT-LA defect. Hemodynamic management can be challenging and involve both pharmacologic as well as mechanical circulatory device therapies. If the defects are hemodynamically significant, definitive therapy involves either interventional or surgical repair.

## Data Availability

No data were used to support this study.
